# Incidence and trends of 17 notifiable bacterial infectious diseases in China, 2004–2019

**DOI:** 10.1186/s12879-023-08194-z

**Published:** 2023-05-02

**Authors:** Yuliang Zhu, Shenghong Lin, Shuaibing Dong, Cuihong Zhang, Lusha Shi, Xiang Ren, Zhongjie Li, Liping Wang, Liqun Fang

**Affiliations:** 1 Division of Infectious Disease, Key Laboratory of Surveillance and Early-warning on Infectious Disease, China CDC, 155 Changbai Road, Changping District, Beijing, China; 2 Central People’s Hospital of Zhanjiang, Zhanjiang, China; 3 Field Epidemiology Training Program, China CDC, Beijing, China; 4 Institute for Infectious Disease and Endemic Disease Control, Beijing Center for Disease Prevention and Control, Beijing Research Center for Preventive medicine, Beijing, China; 5 Fuyang Center for Disease Control and Prevention, Fuyang, China; 6 Complex Systems Research Center, School of Mathematics Sciences, Shanxi University, Taiyuan, China; 7 The State Key Laboratory of Pathogen and Biosecurity, Beijing Institute of Microbiology and Epidemiology, 20 Dong-Da Street, Fengtai District, Beijing, 100071 China

**Keywords:** Bacterial infectious diseases, Joinpoint regression, Incidence, Laboratory confirmation

## Abstract

**Background:**

Certain bacterial infectious diseases are categorized as notifiable infectious diseases in China. Understanding the time-varying epidemiology of bacterial infections diseases can provide scientific evidence to inform prevention and control measures.

**Methods:**

Yearly incidence data for all 17 major notifiable bacterial infectious diseases (BIDs) at the province level were obtained from the National Notifiable Infectious Disease Reporting Information System in China between 2004 and 2019. Of them 16 BIDs are divided into four categories, respiratory transmitted diseases (RTDs, 6 diseases), direct contact/fecal-oral transmitted diseases (DCFTDs, 3 diseases), blood-borne/sexually transmitted diseases (BSTDs, 2 diseases), and zoonotic and vector-borne diseases (ZVDs, 5 diseases), and neonatal tetanus is excluded in the analysis. We characterized the demographic, temporal, and geographical features of the BIDs and examined their changing trends using a joinpoint regression analysis.

**Results:**

During 2004‒2019, 28 779 thousand cases of BIDs were reported, with an annualized incidence rate of 134.00 per 100 000. RTDs were the most commonly reported BIDs, accounting for 57.02% of the cases (16 410 639/28 779 000). Average annual percent changes (AAPC) in incidence were − 1.98% for RTDs, − 11.66% for DCFTDs, 4.74% for BSTDs, and 4.46% for ZVDs. Females had a higher incidence of syphilis than males, and other BIDs were more commonly reported in males. Among 0-5-year-olds, the diseases with the largest increases in incidence were pertussis (15.17% AAPC) and scarlet fever (12.05%). Children and students had the highest incidence rates of scarlet fever, pertussis, meningococcal meningitis, and bacillary dysentery. Northwest China had the highest incidence of RTDs, while South and East China had the highest incidences of BSTDs. Laboratory confirmation of BIDs increased from 43.80 to 64.04% during the study period.

**Conclusions:**

RTDs and DCFTDs decreased from 2004 to 2019 in China, while BSTDs and ZVDs increased during the same period. Great attention should be paid to BSTDs and ZVDs, active surveillance should be strengthened, and timely control measures should be adopted to reduce the incidence.

**Supplementary Information:**

The online version contains supplementary material available at 10.1186/s12879-023-08194-z.

## Introduction

With substantial economic and social development, improvement of the healthcare system, and increased emphasis on prevention, morbidity and mortality from infectious diseases have decreased in China. However, emerging and re-emerging infectious diseases are still a threat to human health and social stability.

The National Notifiable Infectious Disease Reporting Information System (NIDRIS) was launched in the 1950s as China’s sole authorized infectious disease monitoring system. On January 1, 2004, shortly after the 2003 emergence of SARS, China’s government implemented a nationwide Internet-based NIDRIS [1, 2]. NIDRIS currently monitors 40 infectious diseases that are grouped into three categories by pathogen type: bacterial, viral, and parasitic [3] diseases. NIDRIS currently monitors 17 bacterial infectious diseases (BIDs), including plague, cholera, and tuberculosis, etc. (Table S1).

From 1970 to 2007, the annual incidence of infectious diseases in China declined rapidly, from 4000 to 4340 cases per 100,000 in 1970-71 to 120–250 cases per 100,000 in 1990–2007 [4]. A total of 132 858 005 cases of notifiable infectious diseases were reported between 1986 and 2016 - an average annual incidence of 342.14/100 000 [5]. During 2015–2017 there were 121.89 BID cases reported per 100,000 population [6]. Although several studies have explored the epidemic dynamics of notifiable infectious diseases in China, most of them have focused on viral infectious diseases or solely on diseases with a single transmission route, e.g., respiratory infectious diseases [7, 8], and few studies have comprehensively characterized epidemic trends in BID incidence. In this study, based on the national surveillance data on BIDs from 2014 to 2019, we characterize the demographic, temporal, and geographic patterns of BIDs to provide a scientific basis for policymaking, resource allocation, and control and prevention of BIDs.

## Methods

### Data source

Yearly case numbers of 17 BID reports with basic demographic information (sex, age, occupation, region) in the mainland of China during 2004–2019 were extracted from the NIDRIS (China Information System for Disease Control and Prevention, http://219.142.85.3). Annual population sizes for each age group and sex between 2004 and 2019 were retrieved from the NIDRIS for each province.

### Case definitions and classification

A total of 17 notifiable BIDs were included in this study. BIDs are grouped by route of transmission into four categories: respiratory transmitted diseases (RTDs), direct contact/fecal-oral transmitted diseases (DCFTDs), blood-borne/sexually transmitted diseases (BSTDs), and zoonotic and vector-borne diseases (ZVDs). Neonatal tetanus is not included in the categorization and analysis. Medical staff confirmed all patients with a clinical diagnosis by clinical standards and laboratory tests based on national uniform standards and clinically diagnosed and laboratory-confirmed cases were included in this study (Table S1).

### Data analysis

We calculated the number of cases and rates per year for different diseases. We also calculated sex-specific, age-specific (0‒5, 6‒14, 15‒24, 25‒64, 65‒74, ≥ 75 years), region-specific rates (Northeast, North, Northwest, Central, East, South, and Southwest China) of different diseases. We also calculated the proportion of laboratory-confirmed cases per year for different diseases for each year.

Joinpoint regression models were used to examine the changing trends in incidence for each infectious disease from 2004 to 2019 and to estimate annual percentage changes (APCs). The same model was used to test for the differences across regions and sex-age subgroups. Joinpoint regression analysis identifies time points in which trends significantly change (i.e., joinpoints), using calendar year as the timescale.

All analyses were done with R (version 4.0.2) and Joinpoint Regression Analysis program (version 4.8.0.1) (https://surveillance.cancer.gov/joinpoint/). We considered *P* < 0.05 as statistically significant.

## Results

**Fig. 1:**
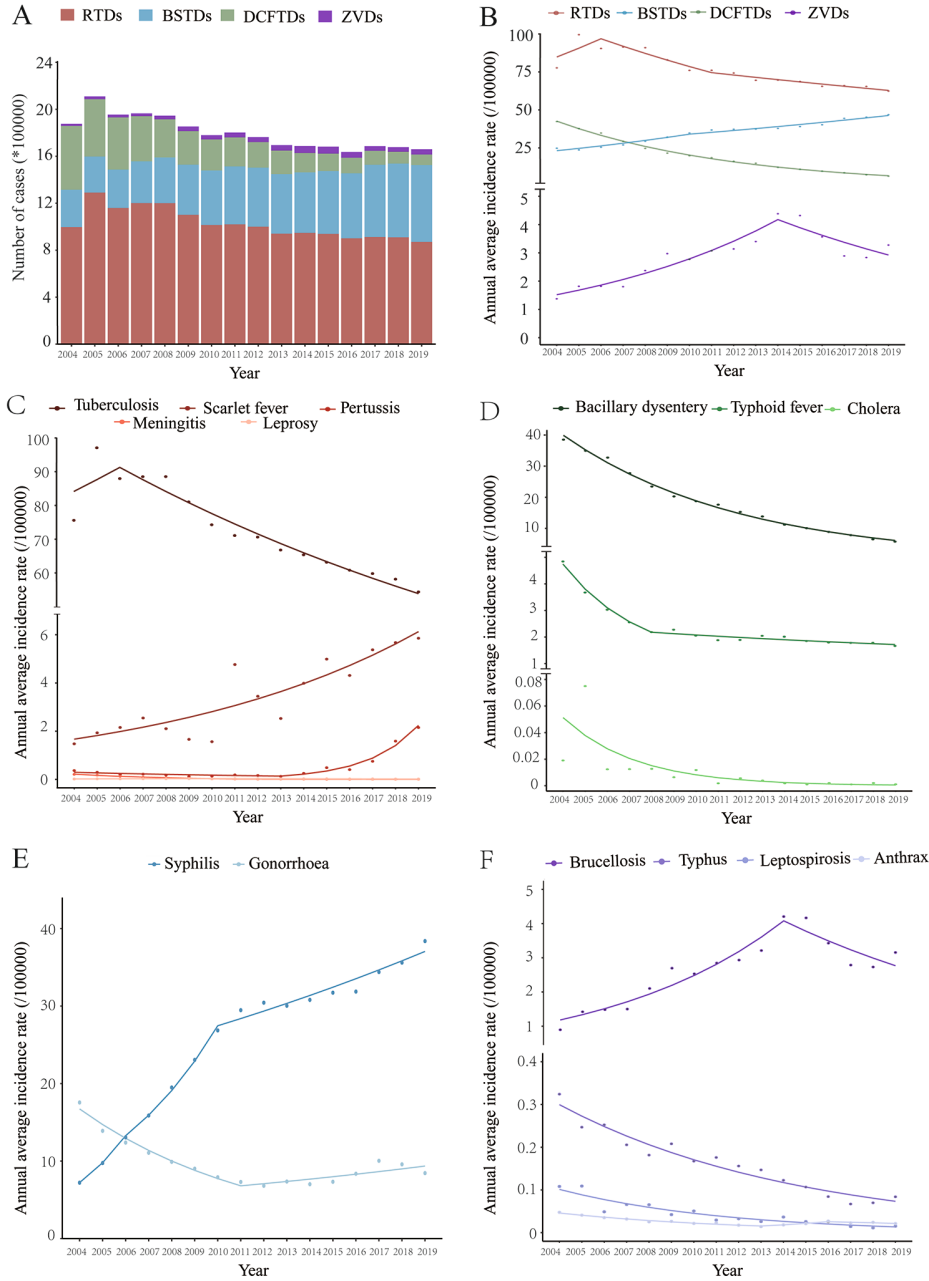
Temporal trend of incidence of the notifiable BIDs by different transmission routes in China, 2004‒2019. **(A)** Number of cases of notifiable BIDs by different transmission routes. There were 28,779,000 BIDs reported; overall AAPC (95%CI) was − 1.87* (-2.56~-1.17); **(B)** Annual average incidence and average annual percentage change (AAPC) of the notifiable bacterial infectious diseases by different transmission route. Respiratory transmitted diseases (RTDs): AAPC (95%CI), -1.98* (-3.08~-0.87); Direct contact/fecal-oral transmitted diseases (DCFTDs), -11.66* (-12.03~-11.29); Blood-borne/sexually transmitted diseases (BSTDs), 4.74* (3.74 ~ 5.74); Zoonotic and vector-borne diseases (ZVDs), 4.46* (1.59 ~ 7.42). **(C)** Annual average incidence and AAPC of RTDs, Tuberculosis, -2.93* (-4.79~-1.03); Scarlet fever, 9.06* (6.47 ~ 11.71); Pertussis, 14.49* (9.12 ~ 20.12); Meningococcal Meningitis, -20.67* (-26.21~-14.72); Leprosy, -0.79 (-3.29 ~ 1.77); **(D)** Annual average incidence and AAPC of DCFTDs, Bacillary dysentery, -11.74* (-12.18~-11.29); Typhoid & paratyphoid fever, -10.45* (-12.23~-8.63); Cholera, -26.32* (-30.77~-21.57). **(E)** Annual average incidence and AAPC of Blood-borne/sexually transmitted diseases (BSTDs), Syphilis, 11.52* (8.82 ~ 14.29); Gonorrhea, -3.80* (-5.73~-1.83). **(F)** Annual average incidence and AAPC of zoonotic and vector-borne diseases (ZVDs), Brucellosis, 5.84* (2.64 ~ 9.14); Typhus, -8.95* (-10.05~-7.83), Leptospirosis, -12.55* (-14.27~-10.79), Anthrax, -4.86 (-9.67 ~ 0.20). Dots indicate the observed incidences and lines indicate slopes of the AAPC.

**Fig. 2:**
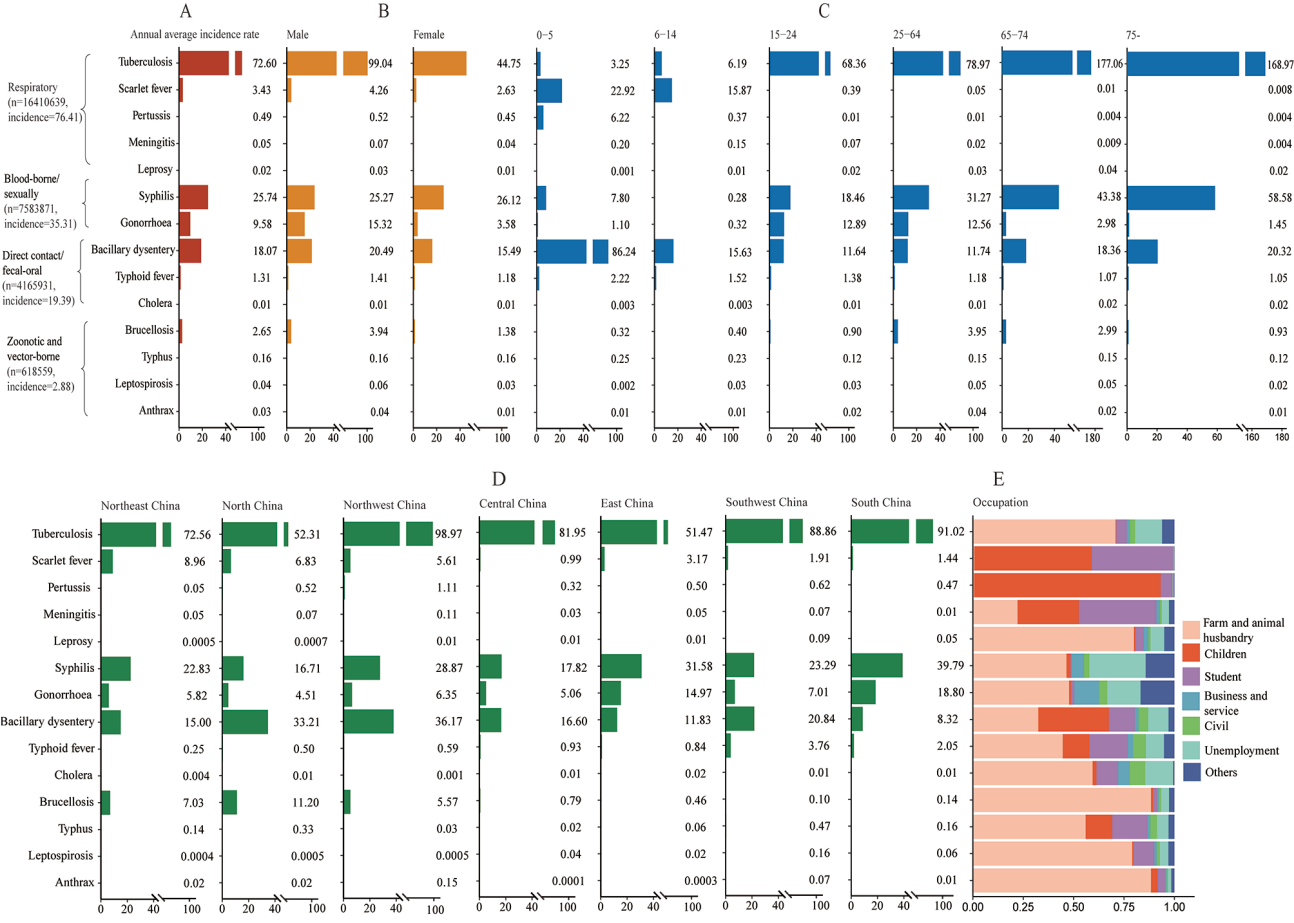
Demographic characteristics of people with reported bacterial infectious diseases. (A) Annual average incidence of bacterial infectious diseases; **(B)** Annual average incidence of bacterial infectious diseases by sex; **(C)** Annual average incidence of bacterial infectious diseases by age; **(D)** Annual average incidence of bacterial infectious diseases by region; **(E)**  The proportion of bacterial infectious diseases by occupation

Between 2004 and 2019, a total of 28 779 thousand BID cases were reported in the mainland of China, and the average annual incidence was 134.00 BID reports per 100,000 population. There was an overall 1.87% decline in BID reports, from 146.18 to 100,000 in 2004 to 118.94 per 100,000 in 2019 (Fig. 1A). By category, RTDs and DCFTDs declined, while BSTDs and ZVDs increased (Fig. 1B). RTDs were the most commonly reported BID during the study period − 16 410 639 cases, accounting for 57.02% of BID reports, for an average annual incidence of 76.41 per 100,000. There were 7 583 871 BSTD cases (35.31 per 100,000), 4 165 931 DCFTD cases (19.39 per 100,000), and 618 559 ZVD cases (2.88 per 100,000) reported during the study period (Figs. 1A and 2 A). RTDs decreased by 1.98% per year (95%CI: 3.08 to 0.87) and DCFTDs decreased by 11.66% (95%CI: 12.03 to 11.29) per year, while BSTDs and ZVDs increased by 4.74% (95%CI: 3.74 to 5.74) and 4.46% (95%CI: 1.59 to 7.42) per year, respectively (Fig. 1B, Table S2).

The average annual incidence of four BIDs - scarlet fever, pertussis, syphilis, and brucellosis - increased significantly from 2004 to 2019, with AAPCs of 9.06%, 14.49%, 11.52%, and 5.84% (Fig. 1C and E F). Pertussis increased the most, with an increase of 59.33% per year between 2013 and 2019. A single inflection point was identified by joinpoint regression for brucellosis (in 2014), with annual increases during 2004‒2014 of 13.20% (95%CI: 9.21 to 17.34) followed by a 7.47% (95%CI: -14.12 to -0.31) decrease per year.

The average annual incidence of cholera, meningococcal meningitis, and leptospirosis decreased the fastest with AAPCs of -26.32% (95%CI: -30.77 to -21.57), -20.67% (95%CI: -26.21 to -14.72) and − 12.55% (95%CI: -14.27 to -10.79), respectively (Fig. 1C and D F).

Although gonorrhea had an AAPC of -3.80% (95%CI: -5.73 to -1.83), a significant increase of 4.06% per year was evident after 2011 (Fig. 1E). The number of plague and diphtheria cases is small, with cumulative reported case counts of 68 and 3 cases.

Tuberculosis and syphilis were the two BIDs with the largest average annual incidences, overall and for males and females separately: 72.60 per 100 000 for tuberculosis and 25.74 per 100 000 for syphilis (Fig. 2A), with males having higher tuberculosis incidence than females and females having a higher incidence of syphilis than males (Fig. 2B). Bacillary dysentery and scarlet fever had the highest incidence among 0‒5-year-olds and 6‒14-year-olds, while tuberculosis and syphilis were predominant in the 15–24, 25–64, 65–74 and ≥ 75 year age groups (Fig. 2C). North and Northwest China had higher incidences of tuberculosis and bacillary dysentery, while other regions had higher incidences of tuberculosis and syphilis (Fig. 2D). Children and students had the higher proportion of scarlet fever, pertussis, meningococcal meningitis, and bacillary dysentery. Other BIDs were more commonly reported among farmers and persons working in animal husbandry (Fig. 2E).

The overall average annual incidence of RTDs increased among 0‒5-year-olds, with an AAPC of 8.10%. Pertussis and scarlet fever had the highest AAPCs at 15.17% and 12.05%, respectively. Among 6‒14-year-olds, AAPCs of syphilis, scarlet fever, and brucellosis were 12.05% (95%CI: 5.83 to 18.63), 9.86% (95%CI: 7.06 to 12.75), and 4.67% (95%CI: 0.28 to 9.26). Although pertussis incidence was stable during 2004‒2019, it increased by 89.93% (95%CI: 59.14 to 126.67) after 2016. Pertussis increased by 17.10% (95%CI: 5.40 to 30.10) among 25‒64-year-olds. Syphilis AAPC was 17.46% (95%CI: 15.67 to 19.28) among individuals 65‒74-year-olds. Among ≥ 75 years, AAPCs of syphilis and brucellosis were 14.66% (95%CI: 11.56 to 17.85), 11.01% (95%CI: 6.26 to 15.98) (Table 1, Table S3).

**Table 1:** Temporal trends of age-specific incidence of bacterial infectious diseases by joinpoint regression analysis, China, 2004–2019 (AAPC, 95%CI).

Bacterial infectious diseases			Age groups				
	0–5	6–14		15–24	25–64	65–74	≥ 75
**Respiratory transmitted diseases** ^**#**^	8.10* (2.31 ~ 14.22)	4.95* (2.92 ~ 7.01)		-3.41* (-4.15~-2.67)	-3.86* (-4.31~-3.39)	-4.33* (-5.10~-3.55)	-4.27* (-6.63~-1.84)
Tuberculosis^a^	-14.16* (-16.02~-12.26)	-3.63* (-5.69~-1.52)		-3.38* (-4.13~-2.63)	-3.86* (-4.32~-3.40)	-4.33* (-5.10~-3.55)	-4.27* (-6.63~-1.84)
Scarlet fever	12.05* (8.80 ~ 15.40)	9.86* (7.06 ~ 12.75)		1.28 (-10.03 ~ 14.01)	0.91 (-0.70 ~ 2.55)	-1.27 (-3.41 ~ 0.91)	-7.40 (-16.64 ~ 2.86)
Pertussis	15.17* (9.22 ~ 21.44)	8.64 (-6.01 ~ 25.57)		3.07 (-7.39 ~ 14.70)	17.10* (5.40 ~ 30.10)	-	-
Meningococcal Meningitis	-18.08* (-24.48~-11.13)	-17.76* (-24.87~-9.97)		-22.45* (-25.94~-18.79)	-24.59* (-27.57~-21.48)	-14.88 (-34.78 ~ 11.09)	-
Leprosy	-	-0.90 (-5.58 ~ 4.00)		-0.87 (-3.68 ~ 2.01)	-1.03 (-3.36 ~ 1.36)	-1.06 (-6.19 ~ 4.34)	-3.77 (-7.49 ~ 0.10)
**Direct contact/fecal-oral transmitted diseases**	-11.56* (-13.02~-10.08)	-12.07* (-14.42~-9.64)		-15.13* (-15.76~-14.50)	-11.86* (-13.58~-10.11)	-10.03* (-11.25~-8.80)	-10.77* (-11.42~-10.12)
Bacillary dysentery	-12.00* (-13.46~-10.52)	-12.39* (-14.92~-9.78)		-15.12* (-15.97~-14.27)	-11.50* (-12.05~-10.96)	-10.34* (-11.67~-8.98)	-11.15* (-12.32~-9.97)
Typhoid & paratyphoid fever	-2.97* (-5.39~-0.48)	-9.78* (-12.21~-7.27)		-14.79* (-17.29~-12.21)	-11.67* (-13.53~-9.77)	-5.26* (-7.70~-2.75)	-3.46 (-7.07 ~ 0.29)
Cholera	-	-		-	-26.19* (-30.93~-21.13)	-	-
**Blood-borne/sexually transmitted diseases**	-4.00* (-6.04~-1.91)	2.29 (-0.33 ~ 4.97)		3.89* (2.82 ~ 4.96)	3.20* (2.67 ~ 3.74)	14.73* (13.51 ~ 15.95)	13.85* (10.86 ~ 16.93)
Syphilis	-1.68 (-4.15 ~ 0.85)	12.05* (5.83 ~ 18.63)		9.03* (7.55 ~ 10.53)	10.13* (7.94 ~ 12.36)	17.46* (15.67 ~ 19.28)	14.66* (11.56 ~ 17.85)
Gonorrhea	-11.23* (-12.76~-9.66)	-3.03 (-6.12 ~0.15)		-0.08 (-2.50 ~ 2.39)	-5.75* (-8.55~-2.86)	-3.16* (-4.91~-1.37)	-3.34* (-4.81~-1.85)
**Zoonotic and vector borne diseases**	0.45 (-2.71 ~ 3.71)	-3.34 (-10.98 ~ 4.97)		-3.34 (-6.71 ~ 0.15)	4.63* (1.65 ~ 7.69)	11.20* (7.58 ~ 14.96)	7.55* (3.74 ~ 11.50)
Brucellosis	8.56* (4.01 ~ 13.32)	4.67* (0.28 ~ 9.26)		-1.40 (-5.22 ~ 2.57)	5.50 *(2.28 ~ 8.82)	12.74* (8.73 ~ 16.89)	11.01* (6.26 ~ 15.98)
Typhus	-9.90* (-12.80~-6.90)	-10.67* (-12.00~-9.31)		-14.95* (-18.41~-11.33)	-8.13* (-9.49~-6.75)	-0.07 (-1.16 ~ 1.02)	-3.63* (-5.50~-1.71)
Leptospirosis	-	-22.82* (-25.37~-20.18)		-18.79* (-24.23~-12.96)	-12.26* (-14.44~-10.03)	-	-
Anthrax	-17.45* (-20.62~-14.16)	-7.28 (-15.01 ~ 1.14)		-4.84* (-8.85 ~ -0.65)	-4.29 (-10.26 ~ 2.07)	-2.44 (-5.28 ~ 0.48)	-3.21 (-10.48 ~ 4.66)

(1) ^a^ indicates that rifampicin resistance cases of tuberculosis in 2017–2019 were removed from AAPC and APC calculations; (2) Neonatal tetanus is not included in the classification by transmission routes; (3) AAPC, average annual percentage change; APC, annual percentage change; 5. * indicate where AAPC or APC differed significantly (p < 0.05) from zero

**Fig. 3:**
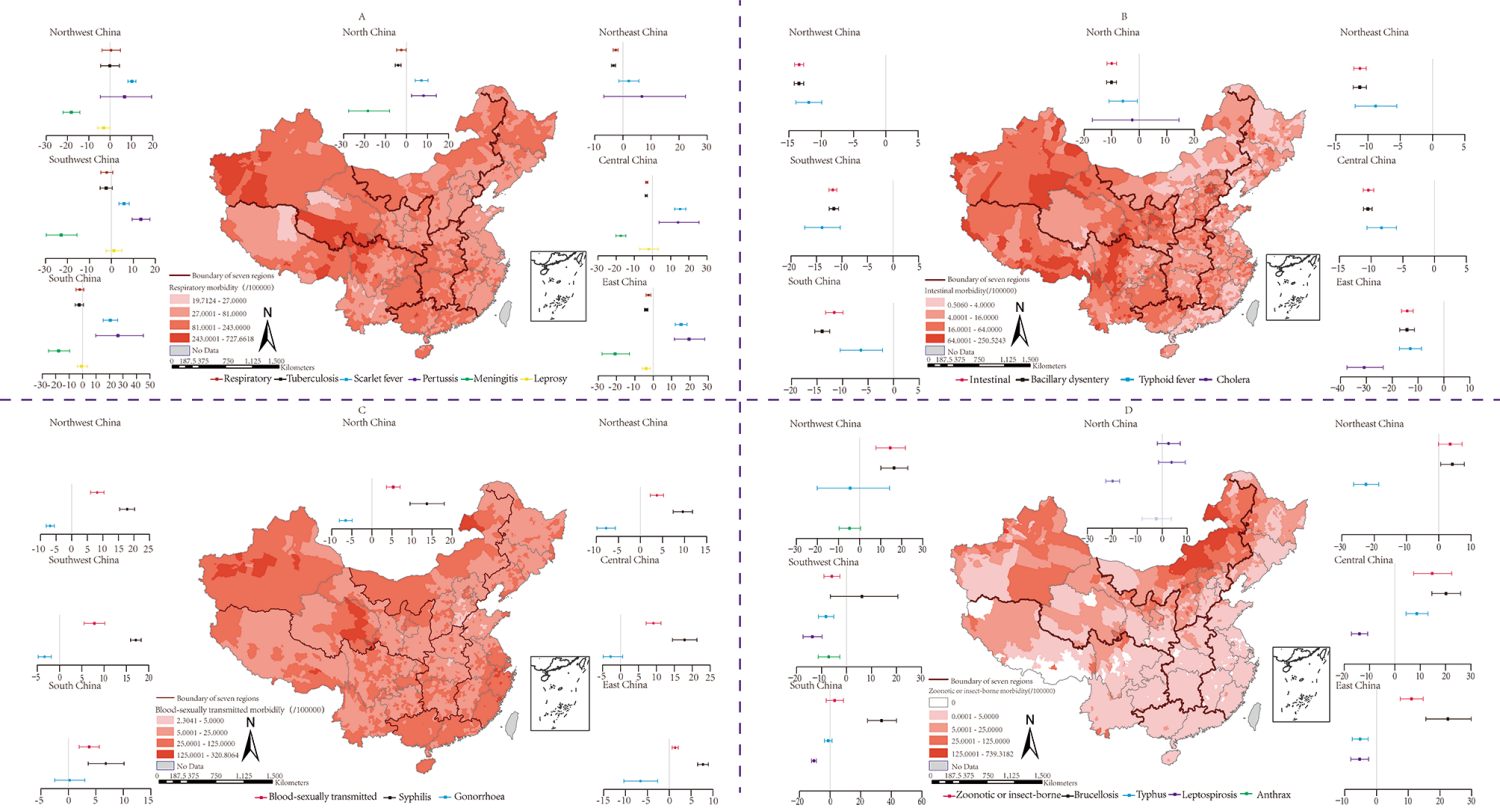
Spatial distribution and temporal trends of BID incidence by disease and region **(A)** Respiratory transmitted diseases (RTDs); **(B)** Direct contact/fecal-oral transmitted diseases (DCFTDs); **(C)** Blood-borne/sexually transmitted diseases (BSTDs); **(D)** zoonotic and vector-borne diseases (ZVDs). The spatial distribution of each category of bacterial infectious diseases is shown in the maps, and the AAPCs of combined and single disease for each category of bacterial infectious diseases is shown in the chart for each region. Uncertainty interval represented around the central dot corresponds to 95% confidence interval

Incidences of RTDs were highest in Northwest and Southwest China counties, while BSTD incidence was highest in coastal and inland border areas of North and Northwest China (Fig. 3A C). DCFTDs incidences were highest in western China and had significant seasonality, peaking in summer and autumn (Fig. 3B, Fig S2). Northern China had the highest incidence of ZVDs (Fig. 3D). DCFTDs decreased in all seven regions, most prominently in East China with an AAPC of -14.19% (95%CI: -16.41 to -11.90). RTDs decreased more slowly than DCFTDs and only in Northeast, North, Central, and East China. BSTDs and ZVDs increased by 9.21% (95%CI: 7.16 ~ 11.31) and 14.68% (95%CI: 7.40 to 22.45) in Central China. Brucellosis, pertussis, and scarlet fever had the most rapid increase in South China, with AAPCs of 33.58% (95%CI: 24.41 to 43.43), 26.27% (95%CI: 9.79 to 45.22), and 20.57% (95%CI: 15.32 to 26.06), respectively. Southwest China had the largest decrease in meningococcal meningitis with an AAPC of -22.90% (95%CI: -29.65~-15.67), while gonorrhea and typhus decreased the most in Northeast China with an AAPCs of -7.80% (95%CI: -9.87~-5.69) and  -22.55% (95%CI: -26.35~-18.55) (Fig. 3, Table S4).

The percentage of BIDs that were laboratory confirmed statistically significantly increased from 43.80% to 2004 to 64.04% in 2019. The average laboratory confirmation rate was 52.22%; laboratory confirmation rates for RTDs (39.38%) and DCFTDs (22.66) were lower than average, while BSTD and ZVD laboratory confirmation rates were higher than average at 93.47 and 87.20%, respectively. Laboratory confirmation rates for syphilis, cholera, and brucellosis were greater than 90%, and laboratory confirmation rates for anthrax and scarlet fever were less than 20% (Table S5).

## Discussion

China’s health sector has made strenuous efforts to improve people’s health over recent decades [25], significantly increasing the strength of public health and the capacity for providing basic medical services and medical security and substantially enhancing physical health and health literacy.

In our study, we determined epidemiological patterns and changes for four categories of bacterial infectious diseases in China by time, region, and population between 2004 and 2019. We found that the incidence of BIDs decreased during the study period, primarily due to reduction in RTDs and DCFTDs. Our data provide evidence that is helpful for clarifying surveillance needs and strengthening BID prevention and control measures.

Although the overall incidence of BIDs decreased, the challenges for BID control are still considerable. Our study showed that tuberculosis still has a high incidence. Northern regions such as Xinjiang had the highest tuberculosis incidence, a phenomenon that may be related to a lower level of economic development and a lack of health education [9]. We also found that the incidence of tuberculosis increased with age, indicating that the elderly were at high risk. To eliminate tuberculosis, strengthening multi-sector cooperation, developing new tuberculosis vaccines, producing new diagnostic and therapeutic drugs, expanding medical insurance coverage, standardizing medical care, and optimizing prevention and treatment strategies are all needed [10].

The incidence of scarlet fever increased fastest among 0‒5- and 6‒14-year-olds, with the highest average annual incidence among six year old children [11]. Children this age are establishing their protective immune functions and may lack immunity against streptococcal infections [12].

Pertussis was decreasing during the first part of the study period, but then began to increase rapidly in all age groups except individuals 65‒74 and over 75-year-olds. One reason for the increase may be waning immunity. Neither natural infection nor vaccination provide lifelong immunity, and people can be infected many times during their life. Vaccine-induced immunity against pertussis is known to wane. Compared with whole-cell pertussis vaccines, acellular pertussis vaccines are less reactogenic and their induced immunity wanes more rapidly [13]. By the second half of the study period, China had switched from whole cell pertussis vaccines to acellular pertussis vaccines. Another potential reason for the increase in pertussis may be evolution of B. pertussis. Emerging B. pertussis lineages (e.g., PtxP3) could be related to the pertussis resurgence [14], as evidenced by the 46.7% prevalence of PtxP3 among isolated circulating strains in Shenzhen [15]. A third possible reason for the increase in pertussis incidence is enhanced B. pertussis surveillance. Medical institutions in many areas have significantly improved their ability and capacity to culture B. pertussis and, especially, to detect pertussis using PCR, resulting in marked improvement in pertussis surveillance sensitivity.

Blood-borne/sexually transmitted diseases incidence increased, with syphilis increasing the most. During 2004‒2006 and 2006‒2010, AAPCs were 35.58% and 19.94%, respectively, but decreased to 3.38% during 2010‒2019. Blood-borne/sexually transmitted diseases incidence, especially syphilis, increased in all seven regions of China, with higher increases in coastal regions and inland border areas of North and Northwest China. As pioneers of economic reform in coastal areas, the rapid development of commercial industries might have led to a higher incidence of syphilis. Sex workers in coastal provinces may have spread syphilis, as has happened in other regions [16]. Syphilis increased the most in relative terms among individuals 65‒74 and over 75-year-olds (AAPC, 17.46%, 14.66%). Syphilis increased in other age groups as well (except for 0-5-year-olds). The increasing incidence of syphilis may also be due to expansion of syphilis screening. Joint screening of HIV and syphilis with voluntary counselling at testing clinics expanded screening, as did preoperative screening for syphilis. Western lifestyles and cultures, including more open discussion about sex, are prominent in Chinese social media [17]. Therefore, Chinese people are more tolerant of a variety of sexual behaviors. In 2010, China promulgated a national syphilis prevention and control program (2010‒2020) [18], which could have been the key driver that slowed the increasing incidence of syphilis that was seen in 2010–2019. The large disease burden of syphilis in China demands more epidemiological investigation, increased screening, expanded partner services, more effective behavioral interventions, and multi-sectoral commitment [19].

Zoonotic and vector-borne diseases incidence increased, mainly due to increases in brucellosis. During 2004‒2014, the incidence of brucellosis increased by 13.20% per year. In the early part of the study period, brucellosis was most prominent in the northern pastureland provinces that practiced animal husbandry and therefore had more opportunity to contact infected livestock. There was also increased attention paid to brucellosis with strengthened brucellosis surveillance. In recent years, transporting animals from different regions led to importation of brucellosis into the southern region [20, 21]. China initiated the National Medium and Long-term Animal Disease Prevention and Control Plan (2012‒2020) and the National Disease Control Plan (2016‒2020), which, together, likely led to the decline in the incidence of brucellosis. After 2014, the incidence decreased by about 7.47% per year. The northern region still has a higher incidence than other regions. To reduce brucellosis, it is important to enhance vaccination of livestock such as cattle and sheep, reduce occupational exposure, and conduct health education for populations at risk of exposure [2].

Etiological diagnosis is essential for clinical management of infectious diseases and for monitoring and providing early warning of infectious diseases [22]. The diagnostic laboratory capacity for infectious diseases in primary medical institutions is insufficient in China. Insufficiency may be due to financial constraints of central medical institutions or a low technical capacity. Therefore, construction of the infectious disease monitoring laboratory network should be further strengthened [23, 24].

Our study had several limitations. First, the data were from NIDRIS, which may lack representativeness with underreporting of BIDs. Second, we did not calculate temporal trends for all BIDs. Age-specific trends of leprosy and cholera could not be calculated with joinpoint regression due to too few cases. Third, multi-drug resistant TBC is an important public health issue. Since July 1, 2017, the “Infectious Disease Reporting Information Management System” has adjusted the TB classification, adding “Rifampicin resistance” to the TB classification. However, due to the small number of reported cases and the short reporting period, no separate study was conducted in this study.

In conclusion, our study showed that incidences of BIDs declined, especially RTDs and DCFTDs, but tuberculosis incidence stayed high. BSTDs and ZVDs increased, but as we adopted control measures against these diseases, the growth in incidence decelerated. We should pay more attention to BIDs, strengthen active monitoring, construct laboratory networks, and adopt timely control measures to reduce the incidence of infectious diseases in China.

## Electronic supplementary material

Below is the link to the electronic supplementary material.

Supplementary Material 1

## Data Availability

The datasets supporting the conclusions of this article are included within the article.

## Abbreviations

AAPC: Overall average annual percent changes
NIDRIS: The National Notifiable Infectious Disease Reporting Information System
BIDs: Bacterial infectious diseases
RTDs: Respiratory transmitted diseases, BSTDs:Blood-borne/sexually transmitted diseases
DCFTDs: Direct contact/fecal-oral transmitted diseases
ZVDs: Zoonotic and vector-borne diseases
